# Enhanced U-Net for Infant Brain MRI Segmentation: A (2+1)D Convolutional Approach

**DOI:** 10.3390/s25051531

**Published:** 2025-02-28

**Authors:** Lehel Dénes-Fazakas, Levente Kovács, György Eigner, László Szilágyi

**Affiliations:** 1 Physiological Controls Research Center, University Research and Innovation Center, Obuda University, 1034 Budapest, Hungary; denes-fazakas.lehel@uni-obuda.hu (L.D.-F.); kovacs@uni-obuda.hu (L.K.); szilagyi.laszlo@uni-obuda.hu (L.S.); 2 Biomatics and Applied Artificial Intelligence Institute, John von Neumann Faculty of Informatics, Obuda University, 1034 Budapest, Hungary; 3 Doctoral School of Applied Informatics and Applied Mathematics, Obuda University, 1034 Budapest, Hungary; 4 Computational Intelligence Research Group, Sapientia Hungarian University of Transylvania, 547366 Targu Mures, Romania

**Keywords:** brain tissue segmentation, MRI data, infant brains, U-net architecture, convolutional neural networks, iSeg-2017 dataset, (2+1)D convolution, medical image processing, deep learning, neural networks

## Abstract

Background: Infant brain tissue segmentation from MRI data is a critical task in medical imaging, particularly challenging due to the evolving nature of tissue contrasts in the early months of life. The difficulty increases as gray matter (GM) and white matter (WM) intensities converge, making accurate segmentation challenging. This study aims to develop an improved U-net-based model to enhance the precision of automatic segmentation of cerebro-spinal fluid (CSF), GM, and WM in 10 infant brain MRIs using the iSeg-2017 dataset. Methods: The proposed method utilizes a U-net architecture with (2+1)Dconvolutional layers and skip connections. Preprocessing includes intensity normalization using histogram alignment to standardize MRI data across different records. The model was trained on the iSeg-2017 dataset, which comprises T1-weighted and T2-weighted MRI data from ten infant subjects. Cross-validation was performed to evaluate the model’s segmentation performance. Results: The model achieved an average accuracy of 92.2%, improving on previous methods by 0.7%. Sensitivity, precision, and Dice similarity scores were used to evaluate the performance, showing high levels of accuracy across different tissue types. The model demonstrated a slight bias toward misclassifying GM and WM, indicating areas for potential improvement. Conclusions: The results suggest that the U-net architecture is highly effective in segmenting infant brain tissues from MRI data. Future work will explore enhancements such as attention mechanisms and dual-network processing for further improving segmentation accuracy.

## 1. Introduction

Brain tissue segmentation using MRI data has remained a subject of extensive investigation for numerous decades, as evidenced by the body of work encompassing studies such as those conducted by Gordillo et al. [1] and Mohan and Subashini [2]. However, when considering the segmentation challenges posed by infant brain tissues, an additional layer of complexity emerges. During the initial months following birth, the T1 data channel of MRI exhibits a notable disparity: the grey matter assumes a lighter hue compared to the white matter. This inherent distinction in coloration aids segmentation. Yet, as the infant reaches six months of age, a remarkable convergence in the intensity distribution of pixels corresponding to these distinct tissue types becomes apparent. The pertinent research conducted by Wang et al. [3] and Sun et al. [4] highlights this intricate overlap.

The advent of automated segmentation methodologies has predominantly aimed to enhance medical workflow by leveraging computational efficiency. Nonetheless, in the specific context of infant brain tissue segmentation, computers are uniquely positioned to discern tissue boundaries that might not be readily discernible to the human eye. Consequently, these computational systems play a pivotal role in facilitating precision decision-making processes.

The exploration of automated segmentation methodologies for infant brain tissue has been a subject of rigorous investigation spanning over a decade. Notably, challenges like iSeg-2017 and iSeg-2019 [3,4] have catalyzed the intensification of these research efforts. These challenges have played a crucial role by not only disseminating meticulously annotated training datasets but also by establishing a standardized framework for benchmarking, thereby fostering advancements in the field.

In the earlier stages, prior to the inception of the iSeg challenges, prevalent methodologies predominantly drew upon classical machine learning techniques [5]. Techniques such as *k*-nearest neighbors, binary decision trees, and support vector machines [6,7] formed the bedrock of these approaches. Additionally, shape models [8,9], along with unsupervised techniques like level sets [10,11] and various *c*-means clustering models [12,13], were frequently employed strategies.

The pivotal emergence and maturation of convolutional neural networks (CNNs) commenced a few years prior to the initiation of the iSeg challenges. Consequently, it is hardly surprising that a significant portion of solutions presented for these challenges, as well as subsequent publications, are rooted in variants of CNNs and deep learning methodologies. Examples of such approaches include works by Bernal et al. [14], Mostapha and Styner [15], Dolz et al. [16]. Furthermore, the utilization of architectures like deep residual networks [17] and U-net networks [18] has become prevalent in the realm of infant brain tissue segmentation, showcasing the rapid integration of cutting-edge neural network architectures into the segmentation process.

Within the ambit of this manuscript, we present a solution catering to the intricate task of infant brain tissue segmentation. Our approach entails the adaptation of a U-net network meticulously aligned with the specific requisites of the problem at hand. Central to our methodology is the utilization of the training dataset consisting of ten records, thoughtfully curated by the experts of the iSeg-2017 challenge.

To enhance the efficacy of our approach, a singular preprocessing step is incorporated into the workflow. This preprocessing step acts as a normalizing mechanism, harmonizing the histograms within both data channels of the MRI records. This crucial normalization process not only aligns the pixel intensities for meaningful comparisons but also sets the stage for subsequent neural network processing. The resultant output of the neural network serves as the ultimate segmentation outcome. To offer an objective appraisal of the segmentation quality, this output undergoes meticulous statistical evaluation, thereby furnishing an unbiased and quantifiable assessment of the achieved results. These pre-processing techniques have already been used in our previous work, so those parts are more at the level of mention. However, the strength of this manuscript is that for the first time, we have applied here a convolutional layer technique used in video processing. In this technique, (2+1)D convolution is used to process the three dimensional space in a step space requesting step. This technique is mainly used in video classification. In the case of our manuscript, we treated the MRI images as if they were videos since the slices are acquired at different moments in time. We did the processing in our neural network with (2+1)D convolutional layers. Moreover, our reference architecture was a U-net solution that we modified. We also achieved our best results so far.

The subsequent sections of this paper are organized as follows: Section 2 presents some previous works of our team upon which the current study relies. Section 3 delves into a comprehensive exposition of both the utilized dataset and the innovative U-net-based segmentation methodology that we proffer. In Section 4, an intricate exploration ensues, where we not only present but also critically discuss the segmentation outcomes that were attained through the employment of our proposed method. Finally, Section 5 encapsulates the denouement of our study, encapsulating key insights drawn and potential avenues for future research.

## 2. Background

In this section, we report some previous solutions and baseline results. Surányi et al. [19] employed classical machine learning techniques to classify the pixels from infant brain MRI volumes based on 18 morphological features and achieved overall accuracy slightly above 83% with the best performing random forest classifier. By the inclusion of atlases for the description of the shape and position of normal brain structures, this segmentation outcome can improve to 84–85% accuracy [20]. However, to be able to compete with the top solutions of the iSeg-2017 challenge, the use of a convolutional neural network is necessary. Unlike classical machine learning approaches that rely on manually crafted features, CNNs automatically learn hierarchical features directly from data. This results in more robust performance and better segmentation accuracy, especially for complex tasks such as infant brain MRI, where subtle structural differences are crucial for reliable classification.

Our first CNN-based solution [21] deployed a simple U-net with 2D convolution with a rather large number of filters. The U-net consisted of four encoder and four decoder blocks and a bridge. The filter numbers for the encoders and decoders were 256, 128, 64, and 32, respectively, while the bridge consisted of 16 filters. The output had four filters, one for each tissue type to be detected and another one for the background pixels. The decoder block consisted of two convolutional layers, two batch normalization layers, a dropout layer, and a max pooling layer. The encoder block also consisted of two convolutional and batch normalization layers and contained a Conv2DTranspose layer that performed the deconvolution. It also contained a concatenate layer and a dropout layer. The bridge was a simplified encoder layer, different in that it did not apply the max pooling operation. For the evaluation, the 10 records took turns in serving as testing dataset, while the other 9 were used as training dataset. The average accuracy achieved by this solution was slightly above 89%.

In a later study [22], we employed a larger 2D U-net. Further on, we changed the cost function, which was now calculated from the sum of the spare categorical cross entropy and the Dice score value, both with a 0.5 weight. We also deployed a U-net with 3D convolution. The structure of the 2D net was similar to the previous one, mainly differing in the filter numbers: 300, 150, 75, and 37, respectively, for the four encoder and decoder blocks, while the bridge contained 18 filters. The 3D convolution U-net also contained four encoders and decoders, with filter counts set to 65, 32, 16, and 8, respectively. The bridge contained four filters. The output also had four classes. Training and testing were performed as in the previous case. The average accuracy achieved by the 2D and 3D U-net models were 90.8% and 91.8%, respectively. These values can compete with the best performing solutions at iSeg challenges [3,4].

## 3. Materials and Methods

### 3.1. U-Net Neural Network Architecture

The U-net neural network architecture has emerged as a pivotal framework in the realm of medical image segmentation, showcasing remarkable capabilities in handling intricate segmentation tasks, including the nuanced challenge of infant brain tissue segmentation. Developed by Ronneberger et al. [23], the U-net architecture stands as a testament to the fusion of architectural innovation and practical effectiveness.

The distinctive structure of the U-net derives its name from its characteristic U-shaped configuration. Comprising two main paths, the network seamlessly integrates both contextual awareness and fine-grained feature extraction. The contracting path, reminiscent of an encoder, initiates the network’s journey by employing a sequence of convolutional layers interspersed with max pooling operations. This phase effectively reduces the spatial dimensions of the input data while meticulously capturing critical features. This process is instrumental in establishing a robust and compact representation of the input image.

Subsequently, the expansive path, akin to a decoder, endeavors to reconstruct the segmented output image from the abstract features generated by the contracting path. Employing transposed convolutional layers, this pathway gradually upscales the features, mirroring the dimensionality reduction of the contracting path. Notably, skip connections play a pivotal role in the U-net’s efficacy. These connections bridge the gap between corresponding layers of the contracting and expansive paths, allowing the network to fuse high-resolution details with context-rich abstractions. This amalgamation of fine-grained and high-level information leads to more precise and contextually grounded segmentation results [23].

The U-net architecture’s success is attributed to its adaptability to various segmentation tasks and its capacity to learn from limited training data. This adaptability has found resonance in the domain of infant brain tissue segmentation, e.g., Dolz et al. [16], Qamar et al. [18]. Its inherent ability to capture intricate spatial relationships, coupled with its capacity to discern between subtle tissue types, positions the U-net as an ideal candidate for tackling the complexities embedded within the infant brain tissue segmentation problem.

In the subsequent sections, we delineate how we have meticulously tailored and configured the U-net architecture to meet the nuanced requirements of infant brain tissue segmentation. Through our customized approach, we leverage the U-net’s strengths to achieve accurate and robust segmentation outcomes.

### 3.2. Dataset and Challenge Context

This study derives its foundation from the meticulously curated training records presented within the iSeg-2017 challenge, an initiative elucidated by Wang et al. [3]. These training records, forming the bedrock of our research, serve as a pivotal resource for the development, validation, and assessment of our proposed U-net-based methodologies.

The iSeg-2017 dataset encompasses a collection of ten distinct records, each of which serves as a reservoir of crucial information for our study. Within each of these records, a multifaceted dataset is meticulously encapsulated. This dataset prominently features T1-weighted and T2-weighted MRI data, acquired from infant subjects. To ensure precise alignment, an automated registration method has been judiciously employed, guaranteeing more or less accurate spatial correspondence between these complementary MRI data modalities. According to the iSeg-2017 challenge organizers, each subject’s T2-weighted volume was rigidly (6-parameter) registered to the corresponding T1-weighted volume prior to distribution. In other words, a standard linear (rigid) registration approach was used so that the T2 dataset matched the coordinate space of the T1. This ensures that both modalities for each subject are spatially consistent and can be compared or fused directly.

Furthermore, an invaluable component of this dataset is the human expert crafted ground truth. This meticulously delineated ground truth annotates pixels corresponding to the three cardinal tissue categories: cerebro-spinal fluid (CSF), gray matter (GM), and white matter (WM). These annotations provide the reference standard against which the segmentation performance of our proposed U-net-based methods will be evaluated.

Each volume within the dataset presents an intricate collection of transversal cross-sections, amounting to no more than 112 slices. These cross-sections are framed by dimensions of 144×192 pixels, effectively capturing the nuanced anatomical details embedded within the infant brain tissues. Noteworthy is the fact that each pixel is representative of a cubic region, measuring one millimeter along each dimension, thus encapsulating crucial information pertaining to the three-dimensional structure of these tissues.

The central issue of the challenge becomes evident when examining the segmentation task as a whole. As infants reach the approximate age of six months, a significant convergence manifests in the intensity distributions of white matter (WM) and gray matter (GM). This results in a perceptual similarity that impedes facile visual differentiation through human observation alone. This inherent ambiguity amplifies the intricacy of the segmentation endeavor, as the intensity-based distinctions between these two pivotal tissue types become indistinct and intertwined [3].

Through a meticulous and systematic exploration of this rich and multifaceted dataset, our study strives to unearth the underlying patterns inherent in infant brain tissue segmentation. In doing so, we aim to contribute to the development of innovative methodologies that adeptly navigate the intricate landscape of infant brain tissue segmentation, particularly in the face of the challenging overlap between white and gray matter tissues.

### 3.3. Data Preprocessing and Intensity Alignment

Within the realm of magnetic resonance imaging (MRI), the pervasive phenomenon of intensity inhomogeneity has long been recognized as a potential source of signal distortion and complexity [24,25]. This low-frequency noise can manifest as irregularities in pixel intensities across the image, casting a veil of uncertainty on subsequent analysis. In the case of our investigation involving the iSeg-2017 training dataset, a meticulous scrutiny reveals that the prevalence of intensity inhomogeneity is notably subdued. Thus, the need for elaborating compensation strategies to counteract this phenomenon is rendered inconsequential for our specific dataset.

Yet, as with any dataset, challenges invariably emerge. In this pursuit, we encounter sporadic gaps and missing data, primarily manifested as voids within specific pixel positions. It is imperative to address these gaps, as their presence can undermine the integrity of subsequent processing and analysis. To mitigate this, we engage in meticulous data augmentation and interpolation techniques, safeguarding the continuity of information flow and preserving the essential context embedded within these spatial regions. We have used histogram-based interpolation.

Beyond these localized gaps, the central issue of our preprocessing endeavor hinges on the harmonization of the diverse intensity scales that inherently characterize the ten distinct MRI records within the iSeg-2017 training dataset. This diversity in intensity distributions can stem from differences in acquisition protocols, imaging hardware, or scanning environments. To harmonize these discrepancies, we invoke a sophisticated preprocessing step entailing histogram alignment.

In realizing this alignment, we embrace a well-established method proposed by Nyúl et al. [26]. This method navigates the intricacies of intensity scale harmonization by leveraging critical percentiles within the intensity distribution. Precisely, we enlist the 25th, 50th, and 75th percentiles as pivotal landmarks within the intensity histograms. By ensuring the alignment of these crucial points across the ten MRI records, we orchestrate a consistent intensity foundation that negates the disparities engendered by the diversity of data acquisition. Importantly, the finesse of our histogram alignment methodology is further informed by insights garnered from prior research, notably presented in our earlier study [27]. This confluence of strategic refinement and established methodology engenders a preprocessing regimen that not only rectifies disparities but also capitalizes on the collective wisdom gleaned from prior research.

Through the meticulous orchestration of these preprocessing measures, our investigation does not merely aim to mitigate data imperfections and promote uniformity; it strives to cultivate a bedrock of data integrity and preprocessing robustness. This foundation, rigorously constructed through a symphony of data augmentation, interpolation, and histogram alignment, poises the dataset for subsequent segmentation analysis driven by the U-net neural network architecture.

### 3.4. Spatiotemporal Convolutions: Unleashing Dynamics in Deep Learning

The evolution of deep learning has ushered in a paradigm shift in our ability to process and understand complex data, and spatiotemporal convolutions stand as a testament to this transformative trajectory. These convolutions, a critical extension of their spatial counterparts, have emerged as a pivotal innovation, enabling the exploration of dynamic patterns and motion information in video data, fundamentally reshaping the landscape of video analysis, action recognition, and beyond.

Spatiotemporal convolutions rise above traditional spatial convolutions by transcending the confines of spatial dimensions and venturing into the temporal dimension, which is indispensable for video data analysis. While spatial convolutions excel at capturing local spatial relationships within single image frames, they fall short in encapsulating the temporal dynamics integral to video sequences. Spatiotemporal convolutions, on the other hand, break through these limitations by seamlessly intertwining spatial and temporal information, elevating deep learning models to comprehend both appearances and motions.

This fusion of spatial and temporal facets is achieved through the simultaneous application of convolutions across three dimensions: two spatial dimensions and one temporal dimension. In practical terms, each spatiotemporal convolutional kernel traverses not only the spatial dimensions but also the timeline of successive frames. This affords the neural network the remarkable ability to discern intricate spatial patterns while simultaneously capturing the temporal evolution of these patterns, effectively encoding movements, actions, and temporal dependencies crucial for tasks like action recognition, video analysis, and gesture understanding.

The utility of spatiotemporal convolutions is perhaps most evident in the domain of action recognition, where precise modeling of the sequence of motions is of paramount importance. By embracing spatiotemporal convolutions, deep neural networks can autonomously and adaptively learn complex spatiotemporal features directly from raw video data, eliminating the need for labor-intensive and domain-specific handcrafted feature engineering.

The architecture of spatiotemporal convolutional networks typically involves the stacking of convolutional layers, often punctuated by pooling and nonlinear activation functions. A pivotal differentiator is the use of 3D convolutional kernels, which stretch across both spatial and temporal dimensions, imbuing the network with the ability to apprehend not only static appearances but also the dynamic flow of actions and events across time.

In the context of deep learning, spatiotemporal convolutions have become emblematic of a powerful synergy between neural networks and temporal dynamics. By enabling the exploration of the spatial and temporal dimensions jointly, these convolutions have proven transformative in domains far beyond action recognition, permeating into fields like video understanding, gesture analysis, and even spatiotemporal anomaly detection.

In closing, the rise of spatiotemporal convolutions has not only broadened the horizons of deep learning but has also nurtured an era of automated understanding of complex temporal data. Their aptitude for harmonizing spatial patterns and temporal dynamics is emblematic of the boundless possibilities that continue to unfold as we harness the potential of deep learning in a world dominated by spatiotemporal data.

**Figure 1:**
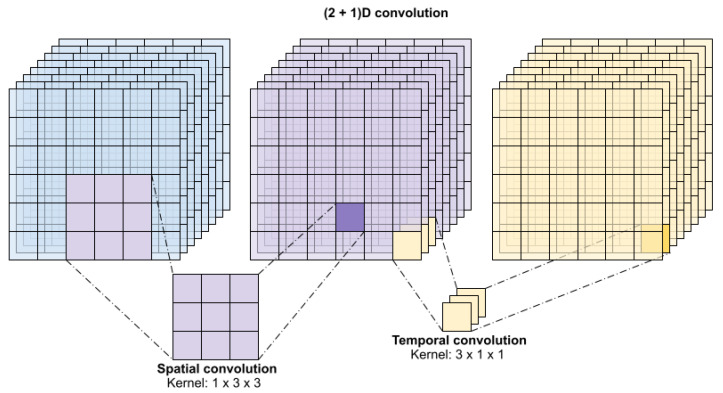
2 plus 1 dimensional convolution.

As is shown in Figure 1, (2+1)D convolutions decompose 3D information into separate spatial and temporal components, potentially causing an imbalance in the learning of spatial and temporal features. As a result, the architecture may struggle to effectively capture the full 3D context, which is crucial for segmenting infant brain tissues characterized by intricate and fine-grained anatomical structures. This limitation could lead to a loss of contextual information along the third dimension, thereby reducing segmentation accuracy, particularly for small or subtle tissue boundaries. I recommend referring to the papers below and exploring boundary-aware loss functions. Refs. [28,29,30] (2+1)D convolutions perform distinct 2D spatial and 1D temporal convolutions, resulting in higher computational costs compared to standard 2D convolutions. Infant brain MRI datasets typically consist of high-resolution 3D volumes, further escalating memory and processing demands. Consequently, training and inference may become impractical on resource-limited systems, restricting their feasibility for clinical applications. However, we treated the input images in three dimensions and not in two dimensions [31,32,33].

**Figure 2:**
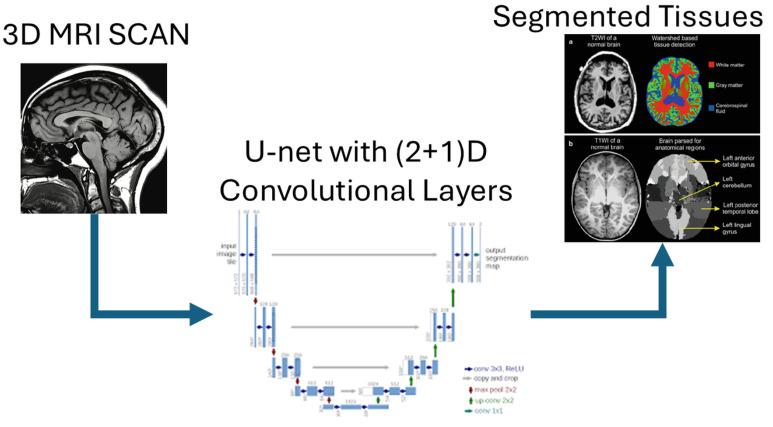
Workflow of our U-net approach. The input is a 3-dimensional MRI scan. This image is passed to our U-net which processes it using (2+1)D convolution. Then, the segmented images are obtained at the output of the last layer. Since all images are segmented output.

Before we go into the details of our network, we would like to demonstrate the workflow of how segmentation works with this network. In order to see the workflow visually we have created a diagram called Figure 2, the input of which is a 3-dimensional MRI image. Next comes our network, which is based on U-net and uses (2+1)D convolution layers. Then, when the network is traversed, we also obtain a 3 dimensional reconstruction with the 3 tissues and the background.

### 3.5. Neural Networks

#### 3.5.1. Overall

**Figure 3:**
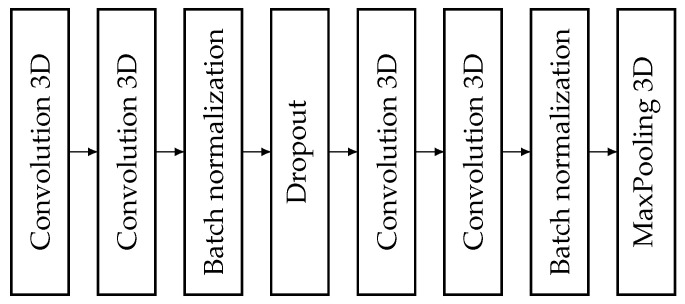
Structure of an encoder block.

Our entire U-net network is built from the experience we have gained so far [21,22], plus we have used the 2 plus 1 dimensional convolutional layer design proposed for video analysis Figure 3. So, our network consists of 4 decoder and encoder parts. In addition, it includes the bridge and skip connections, as well as an input and an output layer. On the input side, since we merged the T1 and T2 channel images, we now have 6 color channels, since we put them next to each other. In addition, we had 112 sections of each volume, and the images were of size 192×144 pixels. This gives us the input size of 112×192×144×6. As it was mentioned before, the encoding part has 4 encoder blocks. The filter numbers are halved at each step, as is the output image size. Therefore, we have the filter numbers in sequence 65, 32 16, and 8. While the image sizes are gradually reducing from 112×192×144, to 56×96×72, then to 28×48×36 and finally to 14×24×18. Of course, the output of each encoder block has a skip connection through to the decoder block situated on the same level. However, this is also an extra output for the encoder block. Next is the bridge with an input size of 7×12×9, the smallest compression in our network. The bridge block uses 4 filters similarly to the output layer. Then comes the resizing, which is carried out by the decoder part. In this branch, similarly to the encoder part, there are 4 decoder blocks. Unlike the encoder, as we go from the bottom up, the filter numbers and the image dimensions increase. So the filter numbers are 8, 16, 32, and 65 in order, while the image sizes are 14×24×18, 28×48×36, 56×96×72 and 112×192×144, respectively. Each decoder block also uses the output of the encoder block situated at the same level, received through the skip connection. Finally, the last layer is the classifier layer with filter number 4, corresponding to the four output classes (three tissue types and outer space). Therefore, our final output has the size of 112×192×144×4.

Table 1 summarizes the layers used in our U-Net network. The main parameters of these layers are ones we have changed. The ones that are not displayed were not changed but left in the default state. In addition, the network was created manually, which means that no transfer learning was applied, as in our other previous research, because in the case of transfer learning, we may not obtain the right result, especially if we use a U-network that was pre-trained on adult data. In the following, we discuss in more detail the structure of the 3 components of the U-net. In addition, there was no data argumentation, as there is so little point in arguing with MRI data. After all, MRI data do not make sense to rotate or flip horizontally or vertically. Therefore, only the raw data were used.

#### 3.5.2. Encoder Blocks

**Figure 4:**
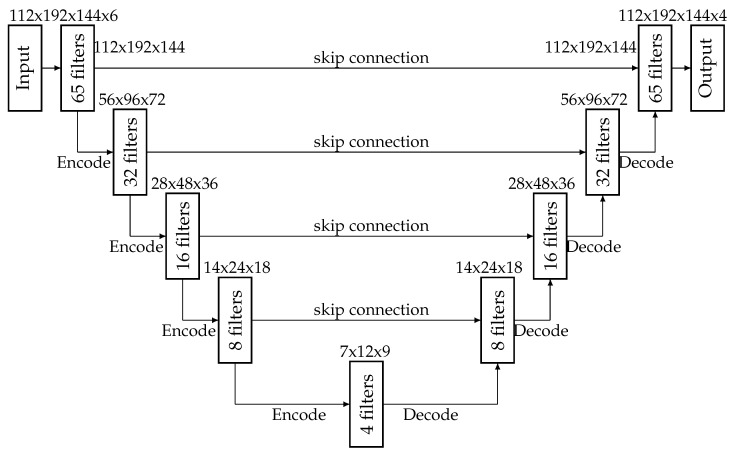
The proposed 2+1D U-net architecture.

The structure of the encoder blocks is exhibited in Figure 3. The encoder blocks contain 4 convolutional layers, 2 batch normalization layers, a dropout layer, and a max pooling layer. The data arriving at the block go first into the first 3D convolutional layer, which is a 3 dimensional convolutional layer, but the kernel is more interesting. Following the rule of 2 plus 1 dimensional operation, and since this is the first convolutional layer, this is computed in space using its kernel. Therefore, the kernel scans an area of 1×3×3. The output of this layer arrives at the next convolutional layer, which is also 3 dimensional. This layer is for spatial feature detection so here, the kernel is 3×1×1, obeying the (2+1)-dimensional convolution rules. The activation function remains ELU [34], as it was found to be the best in our previous studies. The padding property of the convolution layers is set to same. This means that the input and output of the convolution layer have the same size, since they fill in parts of the image with 0 s. Moreover, we used this setting because the dimension reduction is always performed by the max pooling layer. The stride and dilatation attributes were not changed so their values were $\left(1,1,1\right)$. Furthermore, the regularization of the layer was not changed, which means no regularization was used. Instead, it was solved using the Dropout layer. Moreover, the filter number was the number of encoder blocks in each convolution layer in the network, which is the number shown in Figure 4. Next comes a batch normalization layer to normalize the data. Then comes the dropout layer with a rate of 0.2 to avoid overfitting. The dropout layer is followed by two convolutional layers with a similar structure as the first two. The number of filters in the convolutional layers was the same. There was no difference between the number of convolutional layers in a block. The number of filters for each encoding block is indicated in Figure 4. This is followed by another batch normalization layer to normalize the data. Whatever value comes out of this layer will go to the skip connection branch, which then goes to the decoder block, which is on the same level as the encoder. Last comes the max pooling layer, which performs the dimension reduction to halve the volume size. So, the pooling size was set to 2×2×2. Finally, the output is fed one level down to another encoder block or to the bridge.

#### 3.5.3. Bridge

**Figure 5:**
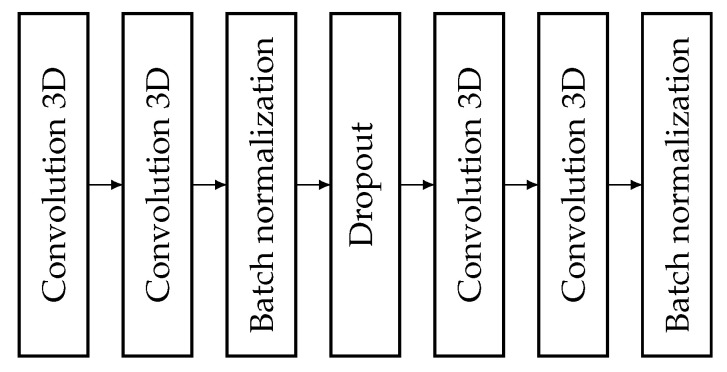
Structure of the bridge part.

The last encoder block is followed by the bridge block in Figure 5. This block connects the encoder part to the decoder part. Furthermore, this block contains the smallest data size that the network works with. Since the U-net is a kind of autoencoder, the bridge can be called the key, the unit that holds the smallest representation. It is similar in structure to an encoder and a decoder, although each block differs in a few ways. The bridge block consists of 4 convolutional layers. In addition, there are two batch normalization layers and a drop out layer. When data arrive in this block, they are passed through the layers. First is a convolutional layer, which is 3 dimensional and has a kernel size of 1×3×3, which is connected to the first convolutional layer of the encoder. This is followed by a batch normalization layer to normalize the data. Then, to avoid over learning, a drop out layer with a value of 0.2 follows. Next are two more 3-dimensional convolution layers. Their kernels are in the familiar form 1×3×3 once and then in the next convolutional layer, they are 3×1×1. Finally, a batch normalization normalizes the data. The convolutional layers use ELU activation and the filter number was already mentioned in Figure 4. All the layers used the same amount with no discrepancies. The convolution layers are configured to use the same padding, ensuring that the input and output dimensions remain the same by zero-padding the input when necessary. This setup guarantees that any dimension reduction occurs in other parts of the network. Moreover, the stride and dilation values were left at their default settings, (1,1,1). Finally, no explicit layer-level regularization (such as weight regularization) was employed.

#### 3.5.4. Decoder

**Figure 6:**
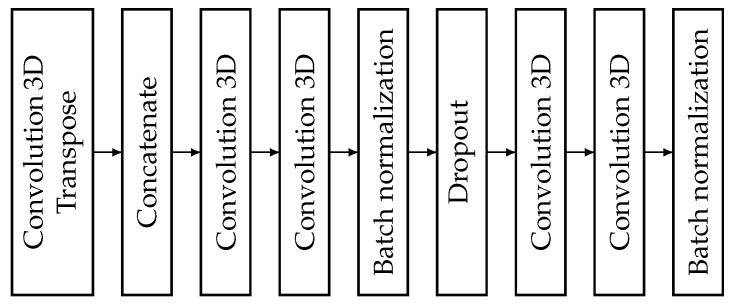
Structure of a decoder block.

Finally, here is the decoder block structure Figure 6. These blocks contain most of the layers, as they use the output of both the encoder block on the same level as them and the output of the bridge or decoder one level down. They contain 4 convolutional layers. Half of these contain two batch normalization layers, as well as containing a conv3dtraspose, concatenate and drop out layer. Let us see step by step how the layers follow each other and how they perform. When data arrive in a decoder block, they first go into the conv3dtraspose layer. These data come from a lower layer, which could be the bridge or another decoder block. This layer is used to increase the dimension of the deconvolution layer. Since the size of the first layer is half the size of the output of the encoder block on that layer, we need to double the size. This is what this layer does. Therefore, it makes sense that the kernel of this layer is 2×2×2, similar to the encoder pooling layer.The fill factor was the same as for other convolution layers. The stride factor was $\left(2,2,2\right)$, in contrast to other convolution layers where it was $\left(1,1,1\right)$. The dilatation was the same. Again, no regularization factor was used here because the dropout layer was used for that. The filter number was the same as the number of decoder blocks in the network. This value can be found in Figure 4. Then comes the concatenate layer, which concatenates the output of the previous layer with the skip connection value at that level. This value comes from the encoder block before dimension reduction. This is followed by two convolution blocks. It is a slap in the face to the previous one. The first convolutional layer searches for features in space and therefore has a kernel size of 1×3×3. While the subsequent convolutional layer, the second convolutional layer, searches for features in time and therefore has a kernel size of 3×1×1. Then follows the batch normalization layer in order to normalize the data. Then follows a dropout layer whose responsibility is to avoid over learning. This is followed again by two convolutional layers that are built in a similar way to the previous ones. That is, the first convolutional layer is 1×3×3 because it searches for features in space, and the second layer is 3×1×1 because it searches for features in time. Finally, there is the batch normalization layer, which is normal on the data. The number of filters in the convolution layer is equal within a block, and their activity is ELU. The convolution layers use the “same” padding so that their input and output dimensions remain identical by zero-padding the input where necessary. This choice is made to ensure that any dimensionality reduction does not occur. The stride and dilation attributes were left at their default values of $\left(1,1,1\right)$. Additionally, no explicit layer-level regularization was applied (i.e., no weight regularization), since overfitting was addressed through a dropout layer. Finally, the number of filters in each convolutional layer corresponds to the number of decoder blocks in the network, as shown in Figure 4.

#### 3.5.5. Classification Layer

The classification layer contains 4 filters, although it is true that our task was to segment the 3 tissue types, i.e., classify them at the pixel level. However, our data also contain a background so that was also marked with a separate class, resulting in four classes. This layer is a 3 dimensional convolutional layer with a kernel axis of 1×1×1 since we are only looking at one pixel at a time. Its activation function is SoftMax, which is well known in classification. The final output that the network gives using this layer is sized 112×192×144×4. That is, for each pixel, we obtain the predicted probabilities that the pixel falls into the four classes. The final segmentation results can be extracted by choosing the highest probability class for each pixel.

### 3.6. Training and Testing

Training and testing was performed in a similar way to our earlier studies, with data from nine infants forming the training dataset at a time and the remaining one infant representing the testing dataset. All this was cross-validated by performing a test for each case, yielding 10 test results. For training, we used our previously proposed cost function [22], which takes into account the sparse categorical cross entropy and the Dice score cost with equal coefficients. The Dice score cost is calculated by subtracting from 1 the average Dice score obtained for the four classes. For the optimization, we used the ADAM optimizer [35]. The network was trained over 1000 epochs in each run. During each training, the best model was saved, which was established by taking the lowest cost function value. For testing, the best model was reloaded, and performance analysis was performed on the test data. Cross validation was carried out one at a time as testing was always performed on one infant. In addition, we used the ADAM optimizer as it is the most widely used optimizer. It also has an adaptive mechanism.

### 3.7. Development Environments

All experiments were conducted using Google Colaboratory (Colab) Pro+, a hosted cloud environment offering enhanced computational resources compared to the free tier. In most sessions, we accessed an NVIDIA A100 GPU with up to 40 GB of dedicated GPU memory, supported by virtualized Intel Xeon CPUs (commonly 2–4 vCPUs) and a high-memory allocation of approximately 25–50 GB of system RAM. This setup runs on a Debian/Ubuntu-based Linux distribution and typically provides around 100 GB of ephemeral storage for data and temporary files.

Within this environment, we rely on Python 3.8 or above (often Python 3.9+) as the primary interpreter. Colab Pro+ includes a notebook-based interface similar to Jupyter Notebook, 6.5.5 enabling interactive execution of code, data analysis, and visualization. Our core libraries include TensorFlow (frequently version 2.13–2.14) for deep learning model development, scikit-learn (version 1.2–1.3) for classical machine learning algorithms and utilities, NumPy (version 1.24–1.25)for numerical computations, and Pandas (version 2.0–2.1) for data manipulation. Additional libraries such as Matplotlib and Seaborn are employed for visualization and exploratory analysis.

For environment management, any additional or updated packages are installed via pip commands within each notebook. Library versions are pinned where necessary (e.g., tensorflow==2.13.0) to maintain consistent experimental conditions across sessions. System resources, including GPU availability, CPU cores, and memory limits, can vary based on usage patterns and resource constraints. Nevertheless, Colab Pro+ generally grants sufficient capacity for efficient training and evaluation of deep learning models, with reduced training times compared to standard free-tier environments. Logs and intermediate results are stored in the ephemeral file system and are periodically backed up to persistent storage (such as Google Drive) to ensure reproducibility and prevent data loss.

This configuration thus provides a flexible, high-performance platform for executing the experiments detailed in this work, leveraging modern deep learning frameworks and a robust Python-based data science ecosystem.

### 3.8. Performance Metrics

The efficacy of the proposed segmentation methodology is meticulously assessed through a comprehensive suite of statistical accuracy indicators. These indicators serve as a quantitative yardstick to gauge the precision, recall overall segmentation performance across different tissue types within the brain.

To formulate the evaluation framework, let us introduce a notation. Denote the set of tissue types within the brain as Ψ={CSF,GM,WM}. Furthermore, for any given record number *i*, let $\Gamma^{\left(i\right)}$ represent the set encompassing all pixels within that record. Additionally, let $\Gamma_{\tau }^{\left(i\right)}$ denote the subset of pixels within record *i* that correspond to tissue type τ, as annotated by the ground truth. The segmentation algorithm assigns pixels to classes, and we denote the set of pixels assigned to class τ within record *i* as $\Lambda_{\tau }^{\left(i\right)}$.

With these definitions in place, we are poised to extract an array of essential statistical indicators, each shedding light on a distinct facet of segmentation quality. For any test volume *i* and any tissue type τ∈Ψ, the following indicators are computed, their definitions detailed in Table 2:

- Sensitivity or True Positive Rate (TPR), also known as Recall: This measures the ratio of correctly identified positive instances to the total actual positives.
- Precision or Positive Predictive Value (PPV): This computes the proportion of true positive predictions among all instances predicted as positive.
- Dice Similarity Score (DSC) or F1-score: This metric captures the balance between precision and recall, providing an overall assessment of segmentation performance.
- Accuracy (ACC): The rate of correct decisions across all tissue types, regardless of their class.

Collectively, these indicators offer a multifaceted view of segmentation performance, encompassing the precision and recall for individual tissue types, as well as the overall accuracy across all tissue types. Each of these statistical accuracy indicators yields a value within the range of 0 to 1, where higher values reflect finer accuracy.

To establish a comprehensive overview of segmentation performance, the overall averages of these indicators are computed based on values derived from individual records. This holistic assessment provides insights into the method’s capacity to accurately delineate brain tissue types, allowing for nuanced comparisons and the identification of strengths and potential areas for improvement.

In conclusion, the ensemble of statistical accuracy indicators furnishes a quantitative lens through which we scrutinize the proposed segmentation methodology’s precision, recall, and overall performance across diverse tissue types within the infant brain.

## 4. Results and Discussion

Let us look at the results we achieved during the tests. Table 3 exhibits the ten infants together with the F1 score, precision, and recall metrics achieved for each in increasing order of the F1 score values. It can be seen that our lowest F1 score value is for infant 4, which is the only case below 91%. That means that even in the worst case, we have 90% accuracy in classifying different brain tissues. In this case, we also achieved the lowest scores for precision and recall. The best result is obtained for infant 8, in which case our F1 score is 2.5% better than the worst case result, giving a score of 93.2%. If we look at the precision and recall scores, we see that both of them reach 93.2%. This is an improvement compared to our previous solutions where we failed to reach 93%. Another interesting fact is that only for four infants did we obtain F1 scores below 92%, which earlier was obtained in six of the ten test cases. Therefore, the improvement is visible from this point of view.

Table 4 presents the accuracy benchmarks obtained for various infants, sorted in increasing order. The bottom row of the table indicated the mean accuracy rate obtained as the average of the accuracy rates of the ten infants, which reaches 92.25%. Accuracy rates obtained for individual infants vary between 90.6% and 93.2%, while the order of the accuracy ranking is identical with the F1-score-based ranking. All our earlier solutions reported at least one infant with accuracy rate below 90%, while the proposed method gives the lowest value of 90.6%. The best accuracy score is obtained for the very same infant 8, who scored best at other benchmarks as well. The average accuracy indicated in the last row is 92.25%, almost half a percent above our previous solution [22].

**Figure 7:**
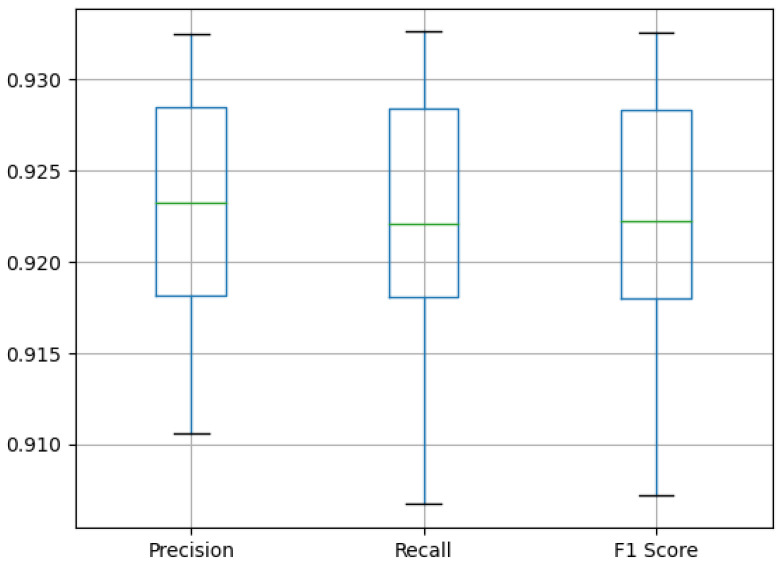
Boxplot of different segmentation benchmark metrics.

Figure 7 exhibits the boxplot of the three benchmark metrics. The median values for all three metrics fall between 92% and 92.5%. This was also inferred from the values in Table 3. Although the medians differ, there is no significant difference among the inter-quartile value (IQR) of all three metrics. Their lower quartile is situated at 91.8%, while their upper quartile is at 92.8%. The lower endpoint for the recall and F1 score values extends below 91%. However, the precision metric does not drop below 91%. Moreover, the upper endpoint is above 93% in all three cases.

Now let us examine the metric values quantified in Table 5. Let us start with the median value, which is 92.3% for precision and 92.2% for recall and F1 score. Thus, we can state that there is a difference of only 0.1% in the median values, which shows that there are no large variances in the tests. This statement is supported by the std row in the table, showcasing values of approximately 0.7%. So there is minimal variance between the test cases, and there are no outliers. Thus, it can be said that the model structure is able to produce the same quality result with minimal difference during the tests. This is further supported by the mean row, where only the precision column shows a significant difference compared to the median. Examining the Q1 row, as mentioned for the boxplot in Figure 7, there was not a big difference, which is now reflected in the numbers. For all three metrics, we obtain 91.8%. This value shows well that we have achieved better results, since this value was the average in the previous solutions. Examining the Q3 line, we have already stated there is not much variation there either among the metrics. As we have already mentioned in the case of the Figure 7 boxplot, we can see that in our best test case, we reached benchmarks above 93%. While in the worst case, we are below 91% in recall and F1 score, we are well above 90% in both cases.

**Figure 8:**
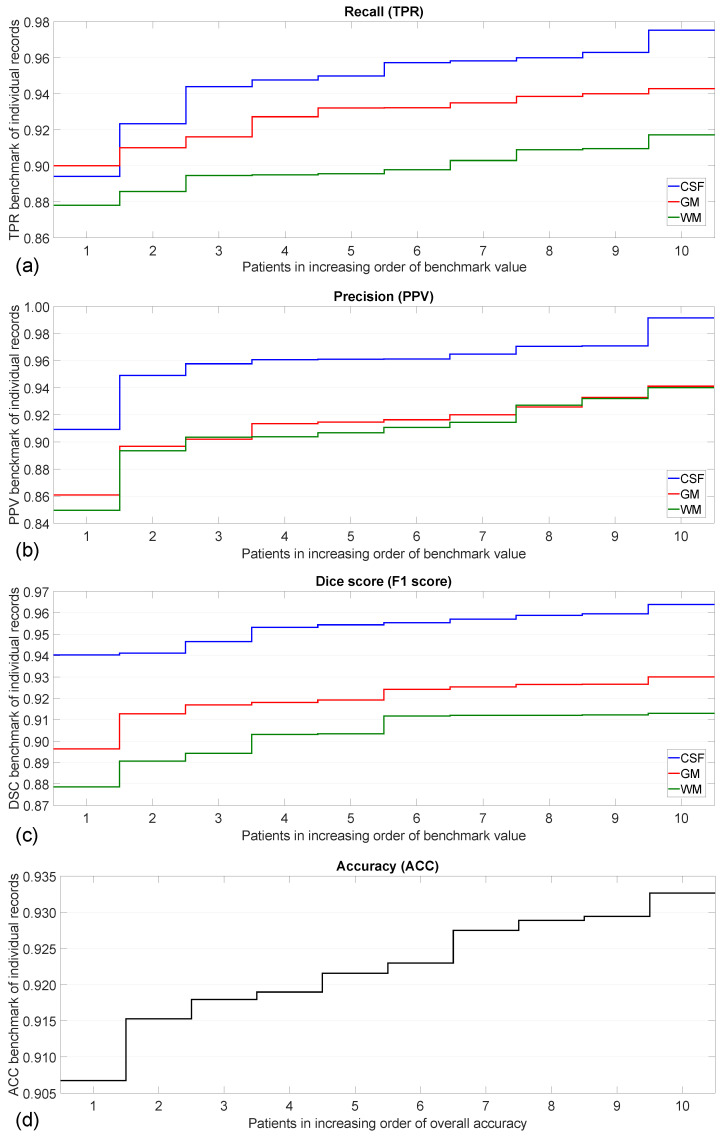
Benchmark values obtained for individual records and tissue types in panels (**a**–**c**); accuracy rates obtained for individual records in panel (**d**).

Figure 8 exhibits the benchmark values obtained for individual records, plotted in increasing order for better visibility. The first three panels show the recall, precision, and F1 score values, respectively, achieved in the case of the three main tissues separately, while the last panel indicated the rate of correct decisions for each record. These graphs indicate that the CSF tissues are identified with best accuracy, which was expected to be so based on the intensity distributions. The recall values are visibly higher for GM than for WM tissues, while the precision is approximately equal for both. The accuracy regardless of tissue type ranges between 90.7% and 93.3%, having a reduced variance around the average value of 92.25%.

Table 6 presents an overview of the confusion matrices obtained for individual records. Furthermore, from this table, it is obvious that the smallest number of misclassifications is related to the CSF tissue. The confusion in CSF and WM is scarce, which does not come as a surprise, knowing the intensity distribution of the tissues in T1 and T2 volumes. The confusion between CSF and GM is more frequent, but the most confusions occur between the GM and WM classes. Approximately the same number of GM pixels are labeled WM as WM pixels labeled GM, which means that the identification of GM pixels is more accurate, because there are almost twice as many GM pixels than WM pixels in the records. This makes the difference that is observable in Figure 8, between F1 scores and recall values of GM and WM tissues.

**Figure 9:**
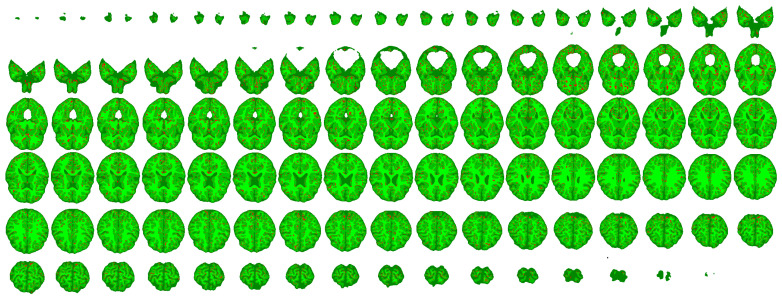
All slices of a segmented brain. The three shades of green from dark to light represent the correctly segmented pixels of the three main tissue types: CSF, GM, and WM, respectively, while red color indicates misclassified pixels.

Figure 9 presents the segmentation outcome of one of the ten records. The three green shades from darker to lighter ones represent the correctly identified pixels of CSF, GM, and WM tissues, respectively. Red color stands for misclassifications, regardless of the tissue types. It is easy to notice that most red pixels are situated at the boundary between two tissue types, where it is not so obvious that the initial annotation was correct.

Table 7 provides a brief performance comparison with our teams previous works and a collection of state-of-the-art methods deployed in the infant brain segmentation process. The improvement with respect to the previous results is obvious and significant, since all benchmark values are higher than in the previous studies. Further on, the column of average F1 scores reveals that the proposed method can compete with the state-of-the-art methods.

## 5. Conclusions

At six months, an infant’s brain is undergoing rapid and dynamic changes, making it an ideal time to investigate emerging neurodevelopmental patterns. Tissue segmentation precisely measures volumes of gray matter, white matter, and cerebrospinal fluid, allowing researchers to track typical maturation trajectories. Subtle anomalies revealed through segmentation can indicate early signs of disorders such as autism spectrum disorder, cerebral palsy, or attention-deficit/hyperactivity disorder. This method also helps clarify the impact of perinatal complications like hypoxic–ischemic encephalopathy or preterm birth on brain structure. Finally, standardized segmentation protocols support meaningful comparisons across studies, leading to improved clinical strategies and interventions. In this paper, we proposed a modified U-net-based method to be employed in the segmentation of infant brain tissues from multi-spectral MRI records. The main novelty in comparison to our previous work was the usage of (2+1)D convolution instead of 3D convolution in all encoder and decoder blocks of the U-net. The proposed method led to improved segmentation quality in comparison to the previous works and can compete with the state-of-the-art methods in all tested statistical benchmarks. Of course, this solution is very limited by the amount of data. Moreover, erroneous annotation may be involved in the fact that the boundary pixels are missed by our model. A further limitation is that we have not yet had the opportunity to test this model in a real environment. We have only tested it on this dataset of 10 infants. It can be seen that robustness is good for these 10 cases, but the dataset needs to be extended to test many more and different data sets. Further improvement could be achieved by extending the proposed model with attention blocks to improve the separation of GM and WM tissues, where the most part of the misclassifications occur. In addition, we like to expand our dataset by obtaining images from the iSeg competition published in other years. This will allow us to gain a larger dataset and also provide cross-validation for testing future models. We also aim to test on data from real hospitals in the future.

## Figures and Tables

**Table 1:** The parameters of each layer of our complete (2+1)d U-network structure that are relevant for the study.

Layer	Filters	Kernel	Strides	Pool	Units	Activation	Dropout
InputLayer	-	-	-	-	-	-	-
Conv3D	65	(1, 3, 3)	(1, 1, 1)	-	-	elu	-
Conv3D	65	(3, 1, 1)	(1, 1, 1)	-	-	elu	-
BN	-	-	-	-	-	-	-
Dropout	-	-	-	-	-	-	0.2
Conv3D	65	(1, 3, 3)	(1, 1, 1)	-	-	elu	-
Conv3D	65	(3, 1, 1)	(1, 1, 1)	-	-	elu	-
BN	-	-	-	-	-	-	-
MPool3D	-	-	(2, 2, 2)	(2, 2, 2)	-	-	-
Conv3D	32	(1, 3, 3)	(1, 1, 1)	-	-	elu	-
Conv3D	32	(3, 1, 1)	(1, 1, 1)	-	-	elu	-
BN	-	-	-	-	-	-	-
Dropout	-	-	-	-	-	-	0.2
Conv3D	32	(1, 3, 3)	(1, 1, 1)	-	-	elu	-
Conv3D	32	(3, 1, 1)	(1, 1, 1)	-	-	elu	-
BN	-	-	-	-	-	-	-
MPool3D	-	-	(2, 2, 2)	(2, 2, 2)	-	-	-
Conv3D	16	(1, 3, 3)	(1, 1, 1)	-	-	elu	-
Conv3D	16	(3, 1, 1)	(1, 1, 1)	-	-	elu	-
BN	-	-	-	-	-	-	-
Dropout	-	-	-	-	-	-	0.2
Conv3D	16	(1, 3, 3)	(1, 1, 1)	-	-	elu	-
Conv3D	16	(3, 1, 1)	(1, 1, 1)	-	-	elu	-
BN	-	-	-	-	-	-	-
MPool3D	-	-	(2, 2, 2)	(2, 2, 2)	-	-	-
Conv3D	8	(1, 3, 3)	(1, 1, 1)	-	-	elu	-
Conv3D	8	(3, 1, 1)	(1, 1, 1)	-	-	elu	-
BN	-	-	-	-	-	-	-
Dropout	-	-	-	-	-	-	0.2
Conv3D	8	(1, 3, 3)	(1, 1, 1)	-	-	elu	-
Conv3D	8	(3, 1, 1)	(1, 1, 1)	-	-	elu	-
BN	-	-	-	-	-	-	-
MPool3D	-	-	(2, 2, 2)	(2, 2, 2)	-	-	-
Conv3D	4	(1, 3, 3)	(1, 1, 1)	-	-	elu	-
Conv3D	4	(3, 1, 1)	(1, 1, 1)	-	-	elu	-
BN	-	-	-	-	-	-	-
Dropout	-	-	-	-	-	-	0.2
Conv3D	4	(1, 3, 3)	(1, 1, 1)	-	-	elu	-
Conv3D	4	(3, 1, 1)	(1, 1, 1)	-	-	elu	-
BN	-	-	-	-	-	-	-
ConvT3d	8	(2, 2, 2)	(2, 2, 2)	-	-	elu	-
Concatenate	-	-	-	-	-	-	-
Conv3D	8	(1, 3, 3)	(1, 1, 1)	-	-	elu	-
Conv3D	8	(3, 1, 1)	(1, 1, 1)	-	-	elu	-
BN	-	-	-	-	-	-	-
Dropout	-	-	-	-	-	-	0.2
Conv3D	8	(1, 3, 3)	(1, 1, 1)	-	-	elu	-
Conv3D	8	(3, 1, 1)	(1, 1, 1)	-	-	elu	-
BN	-	-	-	-	-	-	-
ConvT3d	16	(2, 2, 2)	(2, 2, 2)	-	-	elu	-
Concatenate	-	-	-	-	-	-	-
Conv3D	16	(1, 3, 3)	(1, 1, 1)	-	-	elu	-
Conv3D	16	(3, 1, 1)	(1, 1, 1)	-	-	elu	-
BN	-	-	-	-	-	-	-
Dropout	-	-	-	-	-	-	0.2
Conv3D	16	(1, 3, 3)	(1, 1, 1)	-	-	elu	-
Conv3D	16	(3, 1, 1)	(1, 1, 1)	-	-	elu	-
BN	-	-	-	-	-	-	-
ConvT3d	32	(2, 2, 2)	(2, 2, 2)	-	-	elu	-
Concatenate	-	-	-	-	-	-	-
Conv3D	32	(1, 3, 3)	(1, 1, 1)	-	-	elu	-
Conv3D	32	(3, 1, 1)	(1, 1, 1)	-	-	elu	-
BN	-	-	-	-	-	-	-
Dropout	-	-	-	-	-	-	0.2
Conv3D	32	(1, 3, 3)	(1, 1, 1)	-	-	elu	-
Conv3D	32	(3, 1, 1)	(1, 1, 1)	-	-	elu	-
BN	-	-	-	-	-	-	-
ConvT3d	65	(2, 2, 2)	(2, 2, 2)	-	-	elu	-
Concatenate	-	-	-	-	-	-	-
Conv3D	65	(1, 3, 3)	(1, 1, 1)	-	-	elu	-
Conv3D	65	(3, 1, 1)	(1, 1, 1)	-	-	elu	-
BN	-	-	-	-	-	-	-
Dropout	-	-	-	-	-	-	0.2
Conv3D	65	(1, 3, 3)	(1, 1, 1)	-	-	elu	-
Conv3D	65	(3, 1, 1)	(1, 1, 1)	-	-	elu	-
BN	-	-	-	-	-	-	-
Conv3D	4	(1, 1, 1)	(1, 1, 1)	-	-	softmax	-

**Table 2:** Statistical accuracy indicators with respect to data record *i* and tissue type τ∈Ψ.

Indicator	Definition
Sensitivity	${\mathrm{TPR}}_{\tau }^{\left(i\right)}=\frac{|\Gamma_{\tau }^{\left(i\right)}\cap \Lambda_{\tau }^{\left(i\right)}|}{|\Gamma_{\tau }^{\left(i\right)}|}$
Precision	${\mathrm{PPV}}_{\tau }^{\left(i\right)}=\frac{|\Gamma_{\tau }^{\left(i\right)}\cap \Lambda_{\tau }^{\left(i\right)}|}{|\Lambda_{\tau }^{\left(i\right)}|}$
Dice score	${\mathrm{DSC}}_{\tau }^{\left(i\right)}=\frac{2\cdot |\Gamma_{\tau }^{\left(i\right)}\cap \Lambda_{\tau }^{\left(i\right)}|}{|\Gamma_{\tau }^{\left(i\right)}\left|+\right|\Lambda_{\tau }^{\left(i\right)}|}$
Accuracy	${\mathrm{ACC}}^{\left(i\right)}=\frac{1}{|\Gamma^{\left(i\right)}|}\left|\underset{\tau \in \Psi }{⋃}\left(\Gamma_{\tau }^{\left(i\right)}\cap \Lambda_{\tau }^{\left(i\right)}\right)\right|$

**Table 3:** Recall, precision and F1 score for all infants, displayed in increasing order of the F1 score.

Infant	Precision	Recall	F1 Score
4	0.910571	0.906693	0.907219
7	0.915163	0.914503	0.914542
2	0.917713	0.917772	0.917736
5	0.919483	0.918829	0.918891
10	0.922817	0.921393	0.921721
9	0.923732	0.922788	0.922694
3	0.927507	0.927406	0.927385
6	0.928836	0.928748	0.928642
1	0.929377	0.929290	0.929122
8	0.932538	0.932624	0.932568

**Table 4:** Accuracy benchmark or rate of correct decisions obtained for different infants, sorted in increasing order.

Infant	Accuracy
4	0.906731
7	0.915281
2	0.917950
5	0.918972
10	0.921573
9	0.922976
3	0.927496
6	0.928893
1	0.929426
8	0.932671
**mean**	**0.9225**

**Table 5:** The distribution of various metrics values.

	Precision	Recall	F1 Score
Avg	0.922774	0.922005	0.922052
Std	0.007010	0.007889	0.007732
Min	0.910571	0.906693	0.907219
Q1	0.918155	0.918036	0.918025
Q2	0.923274	0.922090	0.922207
Q3	0.928504	0.928412	0.928328
Max	0.932538	0.932624	0.932568

**Table 6:** Statistics of the ten confusion matrices obtained for individual infants.

		Predicted					
		CSF		GM		WM	
Actual	CSF	Min:	142,971	Min:	3326	Min:	289
		Avg:	166,034	Avg:	9018	Avg:	476
		Sdv:	15,389	Sdv:	4580	Sdv:	198
		Max:	189,750	Max:	20,286	Max:	1029
		Q1:	151,634	Q1:	6412	Q1:	379
		Q2:	168,979	Q2:	7694	Q2:	455
		Q3:	176,538	Q3:	9271	Q3:	475
	GM	Min:	1204	Min:	277,932	Min:	15,867
		Avg:	6199	Avg:	350,375	Avg:	21,216
		Sdv:	2893	Sdv:	31,367	Sdv:	3691
		Max:	13,262	Max:	402,310	Max:	28,401
		Q1:	4769	Q1:	338,884	Q1:	18,309
		Q2:	6045	Q2:	348,180	Q2:	21,157
		Q3:	6979	Q3:	365,025	Q3:	23,837
	WM	Min:	326	Min:	15,842	Min:	162,766
		Avg:	624	Avg:	24,417	Avg:	220,792
		Sdv:	221	Sdv:	5155	Sdv:	32,762
		Max:	996	Max:	34,771	Max:	272,870
		Q1:	462	Q1:	22,572	Q1:	207,567
		Q2:	625	Q2:	23,652	Q2:	219,777
		Q3:	723	Q3:	26,832	Q3:	239,483

**Table 7:** Benchmark comparison with previous works and state-of-the-art methods.

		F1-Score				
Paper	Method	CSF	GM	WM	Average	Accuracy
Surányi et al. [19]	random forest	0.8793	0.8357	0.7963	0.8371	0.8339
Dénes-Fazakas et al. [21]	U-net 2D	0.9272	0.8867	0.8704	0.8948	0.8908
Dénes-Fazakas et al. [22]	U-net 3D	0.9501	0.9152	0.8980	0.9211	0.9180
Proposed method	U-net (2+1)D	0.9528	0.9203	0.9034	0.9255	0.9225
Qamar et al. [18]	U-net 3D	0.9576	0.9205	0.9050	0.9277	N/A
Dolz et al. [16]	CNN ensembles	0.9570	0.9186	0.8971	0.9242	N/A
Nguyen et al. [36]	3D capsules U-net	0.9493	0.9112	0.9021	0.9209	N/A
Tran et al. [37]	self-supervised 3D capsule net	0.9492	0.9148	0.9078	0.9239	N/A

## Data Availability

Access to the data is available upon request. Access to the data can be requested via e-mail to the corresponding author.

## Abbreviations

The following abbreviations are used in this manuscript:

ACC	Accuracy
AI	Artificial intelligence
CNN	Convolutional neural network
CSF	Cerebro-spinal fluid
DSC	Dice similarity score
ELU	Exponential linear unit
GM	Grey matter
IQR	inter-quartile value
MRI	Magnetic resonance imaging
PPV	Precision or positive predictive value
TPR	Sensitivity or true positive rate
WM	White matter
BN	Batch normalization
ConvT3d	ConvT3d
MPool3D	MaxPooling3D
